# Dimethyl 5-nitro­isophthalate

**DOI:** 10.1107/S1600536808024938

**Published:** 2008-08-09

**Authors:** Min-Hao Xie, Pei Zou, Yong-Jun He, Ya-Ling Liu, Biao Huang

**Affiliations:** a Jiangsu Institute of Nuclear Medicine, Wuxi 214063, People’s Republic of China

## Abstract

The nitro group in the title compound, C_10_H_9_NO_6_, is rotated by 10.9 (5)° out of the plane of the benzene ring.

## Related literature

For related literature, see: Bjorsvik *et al.* (2001 ▶); Cutroneo *et al.* (2007 ▶); Enzweiler *et al.* (2006 ▶).
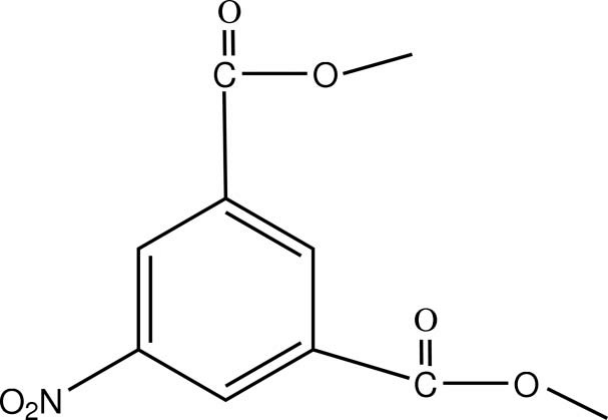


## Experimental

### 

#### Crystal data

C_10_H_9_NO_6_
*M* *_r_* = 239.18
Triclinic, 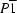

*a* = 4.0130 (8) Å
*b* = 10.660 (2) Å
*c* = 12.643 (3) Å
α = 106.11 (3)°
β = 93.74 (3)°
γ = 91.46 (3)°
*V* = 517.97 (18) Å^3^
*Z* = 2
Mo *K*α radiation
μ = 0.13 mm^−1^
*T* = 293 (2) K
0.40 × 0.05 × 0.05 mm

#### Data collection

Enraf–Nonius CAD-4 diffractometer
Absorption correction: ψ scan (North *et al.*, 1968 ▶)) *T*_min_ = 0.950, *T*_max_ = 0.994
2178 measured reflections
1885 independent reflections
1144 reflections with *I* > 2σ(*I*)
*R*_int_ = 0.041
3 standard reflections every 200 reflections intensity decay: none

#### Refinement

*R*[*F* ^2^ > 2σ(*F* ^2^)] = 0.072
*wR*(*F* ^2^) = 0.172
*S* = 1.01
1885 reflections
154 parameters
H-atom parameters constrained
Δρ_max_ = 0.28 e Å^−3^
Δρ_min_ = −0.18 e Å^−3^

### 

Data collection: *CAD-4 Software* (Enraf–Nonius, 1989 ▶); cell refinement: *CAD-4 Software*; data reduction: *XCAD4* (Harms & Wocadlo, 1995 ▶); program(s) used to solve structure: *SHELXS97* (Sheldrick, 2008 ▶); program(s) used to refine structure: *SHELXL97* (Sheldrick, 2008 ▶); molecular graphics: *SHELXL97*; software used to prepare material for publication: *SHELXL97*.

## Supplementary Material

Crystal structure: contains datablocks I, global. DOI: 10.1107/S1600536808024938/cs2086sup1.cif

Structure factors: contains datablocks I. DOI: 10.1107/S1600536808024938/cs2086Isup2.hkl

Additional supplementary materials:  crystallographic information; 3D view; checkCIF report

## Figures and Tables

**Fig. 1.**
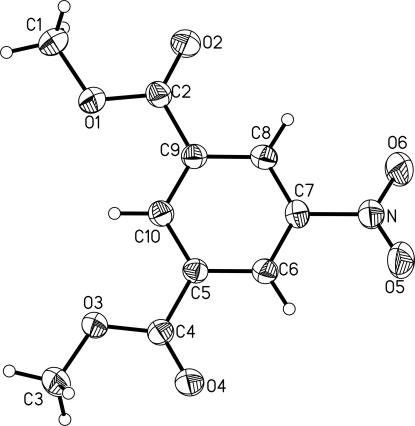
A view of the title compound with the atomic numbering scheme. Displacement ellipsoids were drawn at the 30% probability level.

## supplementary crystallographic information

### Comment

The title molecule (Fig.1), is useful as an important intermediate for the preparation of iodinated *X*-ray contrast media, in particular non-ionic ones such as Iopamidol, Iohexol and Ioversol, being used clinically all over the world (Cutroneo *et al.*, 2007; Bjorsvik *et al.*, 2001; Enzweiler *et al.*, 2006). This crystal structure shows that the benzene ring and the nitro group are only slightly inclined, as shown by the torsion angles of O5—N—C7—C6 -10.6 (5)° and of O6—N—C7—C8 -11.2 (5)°.

### Experimental

5-nitroisophthalic acid (0.5 mmol,100.6 mg) was dissolved in hot methanol (5 ml), then a drop of concentrated sulfuric acid was added and refluxed for 4 h. The precipitate was filtered off, washed with water and dissolved in 95% ethanol(20 mL). The solution was evaporated in air affording colourless needle crystals suitable for X-ray analysis (yield: 85.2%).

### Refinement

Positional parameters of all the H atoms bonded to C atoms were calculated geometrically and were allowed to ride on the C atoms to which they are bonded, with C—H = 0.93 (aromatic) and with *U*_iso_(H) = 1.2U_eq_(C) or 0.96 (methyl) and *U*_iso_(H) = 1.5U_eq_(C), respectively.

### Crystal data

**Table:**

C_10_H_9_NO_6_	*Z* = 2
*M**_r_* = 239.18	*F*_000_ = 248
Triclinic, *P*1	*D*_x_ = 1.534 Mg m^−^^3^
Hall symbol: -P 1	Mo *K*α radiation λ = 0.71073 Å
*a* = 4.0130 (8) Å	Cell parameters from 25 reflections
*b* = 10.660 (2) Å	θ = 8–12º
*c* = 12.643 (3) Å	µ = 0.13 mm^−^^1^
α = 106.11 (3)º	*T* = 293 (2) K
β = 93.74 (3)º	Needle, colourless
γ = 91.46 (3)º	0.40 × 0.05 × 0.05 mm
*V* = 517.97 (18) Å^3^	

### Data collection

**Table:**

Enraf–Nonius CAD-4 diffractometer	*R*_int_ = 0.041
Radiation source: fine-focus sealed tube	θ_max_ = 25.3º
Monochromator: graphite	θ_min_ = 1.7º
*T* = 293(2) K	*h* = −4→4
ω/2θ scans	*k* = −12→12
Absorption correction: ψ scan(North *et al.*, 1968))	*l* = 0→15
*T*_min_ = 0.950, *T*_max_ = 0.994	3 standard reflections
2178 measured reflections	every 200 reflections
1885 independent reflections	intensity decay: none
1144 reflections with *I* > 2σ(*I*)	

### Refinement

**Table:**

Refinement on *F*^2^	Secondary atom site location: difference Fourier map
Least-squares matrix: full	Hydrogen site location: inferred from neighbouring sites
*R*[*F*^2^ > 2σ(*F*^2^)] = 0.073	H-atom parameters constrained
*wR*(*F*^2^) = 0.172	*w* = 1/[σ^2^(*F*_o_^2^) + (0.05*P*)^2^ + 0.6*P*] where *P* = (*F*_o_^2^ + 2*F*_c_^2^)/3
*S* = 1.01	(Δ/σ)_max_ < 0.001
1885 reflections	Δρ_max_ = 0.28 e Å^−^^3^
154 parameters	Δρ_min_ = −0.18 e Å^−^^3^
Primary atom site location: structure-invariant direct methods	Extinction correction: none

### Fractional atomic coordinates and isotropic or equivalent isotropic displacement parameters (Å^2^)

**Table:**

	*x*	*y*	*z*	*U*_iso_*/*U*_eq_	
N	0.5209 (9)	0.7040 (3)	0.8012 (3)	0.0572 (8)	
O1	0.7307 (7)	0.1642 (2)	0.5059 (2)	0.0642 (8)	
C1	0.8443 (12)	0.0907 (4)	0.4027 (3)	0.0717 (13)	
H1A	0.8215	−0.0010	0.3965	0.108*	
H1B	1.0748	0.1145	0.3992	0.108*	
H1C	0.7126	0.1094	0.3431	0.108*	
O2	0.8638 (9)	0.3487 (3)	0.4668 (2)	0.0825 (10)	
C2	0.7532 (9)	0.2943 (3)	0.5284 (3)	0.0480 (9)	
O3	0.2103 (7)	0.1601 (2)	0.8322 (2)	0.0608 (8)	
C3	0.0691 (12)	0.0824 (4)	0.8961 (4)	0.0703 (12)	
H3A	0.0833	−0.0086	0.8583	0.105*	
H3B	−0.1610	0.1022	0.9054	0.105*	
H3C	0.1906	0.1016	0.9671	0.105*	
O4	0.1018 (8)	0.3426 (3)	0.9595 (2)	0.0739 (9)	
C4	0.2108 (9)	0.2883 (3)	0.8735 (3)	0.0482 (9)	
C5	0.3639 (8)	0.3588 (3)	0.8013 (3)	0.0424 (8)	
O5	0.3683 (10)	0.7593 (3)	0.8783 (3)	0.0957 (12)	
C6	0.3703 (9)	0.4947 (3)	0.8341 (3)	0.0465 (9)	
H6A	0.2839	0.5401	0.8996	0.056*	
O6	0.6840 (9)	0.7614 (3)	0.7524 (3)	0.0910 (11)	
C7	0.5090 (8)	0.5598 (3)	0.7663 (3)	0.0445 (8)	
C8	0.6352 (8)	0.4972 (3)	0.6687 (3)	0.0457 (8)	
H8A	0.7276	0.5447	0.6254	0.055*	
C9	0.6236 (9)	0.3612 (3)	0.6351 (3)	0.0448 (8)	
C10	0.4871 (8)	0.2942 (3)	0.7022 (3)	0.0450 (8)	
H10A	0.4781	0.2033	0.6802	0.054*	

### Atomic displacement parameters (Å^2^)

**Table:**

	*U*^11^	*U*^22^	*U*^33^	*U*^12^	*U*^13^	*U*^23^
N	0.065 (2)	0.0474 (18)	0.061 (2)	0.0053 (16)	0.0112 (17)	0.0155 (16)
O1	0.091 (2)	0.0508 (15)	0.0511 (15)	0.0022 (14)	0.0238 (14)	0.0100 (12)
C1	0.099 (4)	0.062 (2)	0.054 (2)	0.013 (2)	0.027 (2)	0.009 (2)
O2	0.124 (3)	0.0604 (17)	0.0677 (19)	−0.0108 (17)	0.0448 (18)	0.0181 (15)
C2	0.049 (2)	0.0464 (19)	0.050 (2)	−0.0043 (16)	0.0047 (17)	0.0156 (17)
O3	0.085 (2)	0.0465 (14)	0.0549 (16)	0.0031 (13)	0.0257 (14)	0.0166 (12)
C3	0.089 (3)	0.057 (2)	0.072 (3)	−0.002 (2)	0.027 (2)	0.025 (2)
O4	0.105 (2)	0.0593 (17)	0.0644 (18)	0.0145 (16)	0.0411 (17)	0.0209 (14)
C4	0.049 (2)	0.049 (2)	0.048 (2)	0.0061 (16)	0.0103 (17)	0.0131 (17)
C5	0.0417 (19)	0.0443 (18)	0.0420 (19)	0.0034 (15)	0.0021 (15)	0.0139 (15)
O5	0.134 (3)	0.0523 (17)	0.107 (3)	0.0219 (18)	0.057 (2)	0.0198 (17)
C6	0.050 (2)	0.0473 (19)	0.0437 (19)	0.0042 (16)	0.0029 (16)	0.0145 (16)
O6	0.131 (3)	0.0527 (17)	0.089 (2)	−0.0210 (18)	0.036 (2)	0.0148 (16)
C7	0.0388 (19)	0.0405 (17)	0.054 (2)	0.0001 (15)	−0.0011 (16)	0.0137 (16)
C8	0.045 (2)	0.0507 (19)	0.0432 (19)	−0.0005 (16)	0.0012 (16)	0.0174 (16)
C9	0.047 (2)	0.0464 (19)	0.0412 (18)	0.0013 (16)	0.0028 (16)	0.0127 (15)
C10	0.044 (2)	0.0449 (18)	0.046 (2)	−0.0032 (15)	0.0042 (16)	0.0130 (16)

### Geometric parameters (Å, °)

**Table:**

N—O6	1.194 (4)	C3—H3B	0.9600
N—O5	1.204 (4)	C3—H3C	0.9600
N—C7	1.475 (4)	O4—C4	1.198 (4)
O1—C2	1.336 (4)	C4—C5	1.485 (5)
O1—C1	1.433 (4)	C5—C10	1.381 (4)
C1—H1A	0.9600	C5—C6	1.391 (5)
C1—H1B	0.9600	C6—C7	1.378 (5)
C1—H1C	0.9600	C6—H6A	0.9300
O2—C2	1.193 (4)	C7—C8	1.366 (5)
C2—C9	1.475 (5)	C8—C9	1.392 (5)
O3—C4	1.320 (4)	C8—H8A	0.9300
O3—C3	1.438 (4)	C9—C10	1.381 (5)
C3—H3A	0.9600	C10—H10A	0.9300
			
O6—N—O5	122.5 (3)	O4—C4—C5	123.3 (3)
O6—N—C7	118.6 (3)	O3—C4—C5	112.4 (3)
O5—N—C7	118.9 (3)	C10—C5—C6	119.7 (3)
C2—O1—C1	116.9 (3)	C10—C5—C4	122.3 (3)
O1—C1—H1A	109.5	C6—C5—C4	117.9 (3)
O1—C1—H1B	109.5	C7—C6—C5	117.8 (3)
H1A—C1—H1B	109.5	C7—C6—H6A	121.1
O1—C1—H1C	109.5	C5—C6—H6A	121.1
H1A—C1—H1C	109.5	C8—C7—C6	123.1 (3)
H1B—C1—H1C	109.5	C8—C7—N	118.7 (3)
O2—C2—O1	122.5 (3)	C6—C7—N	118.2 (3)
O2—C2—C9	124.5 (3)	C7—C8—C9	119.0 (3)
O1—C2—C9	112.9 (3)	C7—C8—H8A	120.5
C4—O3—C3	116.9 (3)	C9—C8—H8A	120.5
O3—C3—H3A	109.5	C10—C9—C8	118.7 (3)
O3—C3—H3B	109.5	C10—C9—C2	122.6 (3)
H3A—C3—H3B	109.5	C8—C9—C2	118.7 (3)
O3—C3—H3C	109.5	C5—C10—C9	121.6 (3)
H3A—C3—H3C	109.5	C5—C10—H10A	119.2
H3B—C3—H3C	109.5	C9—C10—H10A	119.2
O4—C4—O3	124.3 (3)		
			
C1—O1—C2—O2	−0.4 (6)	O6—N—C7—C6	168.8 (4)
C1—O1—C2—C9	178.7 (3)	O5—N—C7—C6	−10.6 (5)
C3—O3—C4—O4	−0.1 (6)	C6—C7—C8—C9	0.2 (5)
C3—O3—C4—C5	179.7 (3)	N—C7—C8—C9	−179.7 (3)
O4—C4—C5—C10	−179.8 (4)	C7—C8—C9—C10	−0.6 (5)
O3—C4—C5—C10	0.4 (5)	C7—C8—C9—C2	178.6 (3)
O4—C4—C5—C6	−2.1 (5)	O2—C2—C9—C10	177.7 (4)
O3—C4—C5—C6	178.1 (3)	O1—C2—C9—C10	−1.4 (5)
C10—C5—C6—C7	−1.5 (5)	O2—C2—C9—C8	−1.4 (6)
C4—C5—C6—C7	−179.3 (3)	O1—C2—C9—C8	179.5 (3)
C5—C6—C7—C8	0.8 (5)	C6—C5—C10—C9	1.2 (5)
C5—C6—C7—N	−179.2 (3)	C4—C5—C10—C9	178.9 (3)
O6—N—C7—C8	−11.2 (5)	C8—C9—C10—C5	−0.1 (5)
O5—N—C7—C8	169.4 (4)	C2—C9—C10—C5	−179.3 (3)

## References

[bb1] Bjorsvik, H.-R., Priebe, H., Cervenka, J., Aabye, A. W., Gulbrandsen, T. & Bryde, A. C. (2001). *Org. Process Res. Dev.* **5**, 472–478.

[bb2] Cutroneo, P., Polimeni, G., Curcuruto, R., Calapai, G. & Caputi, A. P. (2007). *Pharmacol. Res.* **56**, 35–41. 10.1016/j.phrs.2007.03.003 17482832

[bb3] Enraf–Nonius (1989). *CAD-4 Software* Enraf–Nonius, Delft, The Netherlands.

[bb4] Enzweiler, C. N. H., Höhn, S., Taupitz, M., Lembcke, A. E., Wiese, T. H., Hamm, B. & Kivelitz, D. E. (2006). *Acad. Radiol.* **13**, 95–103. 10.1016/j.acra.2005.09.089 16399037

[bb5] Harms, K. & Wocadlo, S. (1995). *XCAD4* University of Marburg, Germany.

[bb6] North, A. C. T., Phillips, D. C. & Mathews, F. S. (1968). *Acta Cryst.* A**24**, 351–359.

[bb7] Sheldrick, G. M. (2008). *Acta Cryst.* A**64**, 112–122. 10.1107/S0108767307043930 18156677

